# Exercise Outcomes in Childhood Obesity-Related Inflammation and Oxidative Status

**DOI:** 10.3389/fnut.2022.886291

**Published:** 2022-07-04

**Authors:** Brisamar Estébanez, Chun-Jung Huang, Marta Rivera-Viloria, Javier González-Gallego, María J. Cuevas

**Affiliations:** ^1^Institute of Biomedicine (IBIOMED), University of León, León, Spain; ^2^Exercise Biochemistry Laboratory, Department of Exercise Science and Health Promotion, Florida Atlantic University, Boca Raton, FL, United States; ^3^Centro de Investigación Biomédica en Red de Enfermedades Hepáticas y Digestivas (CIBERehd), Madrid, Spain

**Keywords:** adipokines, antioxidant systems, cytokines, children, exercise, inflammation, oxidative stress, physical activity

## Abstract

Childhood obesity is identified as one of the major public health issues to increase the risk for cardiometabolic diseases and related complications in adulthood. The literature has supported inflammation and oxidative stress as the primary underlying mechanisms involved in the pathogenesis of obesity-related diseases. Epidemiological evidence consistently shows the benefits of physical activity in the improvement of obesity-mediated inflammation and oxidative stress status. In this narrative mini-review, the available scientific evidence on the potential effects of exercise in alleviating these susceptibilities in childhood obesity will be assessed.

## Introduction

**FIGURE 1:**
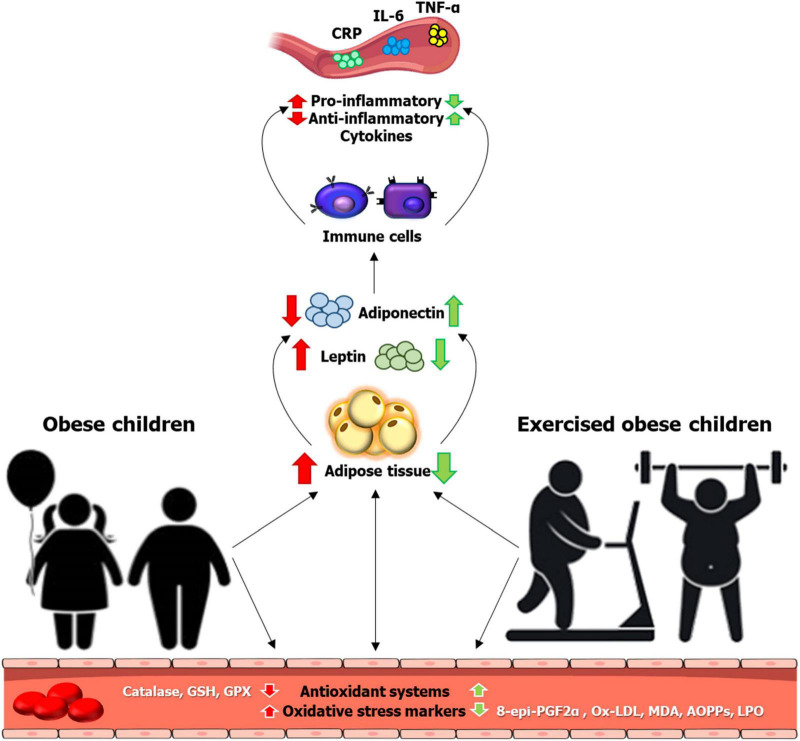
Inflammation and oxidative stress responses to childhood obesity and exercise in obese children. Localized inflammation in adipose tissue related to obesity triggers low-grade systemic inflammation, manifesting in a general increase in circulating inflammatory markers in obese children. Childhood obesity concomitates with an oxidative status imbalance, increasing the oxidative stress markers and decreasing the antioxidant systems. Different protocols of physical activity might revert the consequences of obesity on inflammatory processes and oxidative stress during child growth. 8-epi-PGF2α, 8-epi-prostaglandin F2 alpha; AOPPs, advanced oxidation protein products; CRP, C-reactive protein; GPX, glutathione peroxidase; GSH, reduced glutathione; IL-6, interleukin 6; LPO, lipoperoxides; MDA, malonaldehyde; ox-LDL, oxidized low-density lipoproteins; TNF-α, tumor necrosis factor alpha.

Childhood obesity is considered as one of the most important public health issues, with approximately 5 times more likely to develop obesity in adulthood (1). Obesity-attributable illnesses, such as cardiovascular disease have contributed to more than two-thirds of deaths globally, according to data from the Global Burden of Disease study (2). One of the underlying mechanisms contributing to obesity-related diseases is the development of systemic low-grade inflammation derived from adipose tissue (3, 4). While chronic inflammation is often associated with increased oxidative stress (5), the literature has previously reported the role of oxidative stress in the pathogenesis of obesity-related diseases (6). Other possible contributors to elevated oxidative stress in obesity include, but not limited to, hyperglycemia, vitamin and mineral deficiencies, hyperleptinemia, endothelial dysfunction, and impaired mitochondrial function (7). Intricately, the damage of cellular proteins by reactive oxygen species (ROS) triggers the inflammatory responses to target the pattern recognition receptors, located either on the cell membrane [e.g., Toll-like receptors (TLRs)] (8) or in the cytoplasm [e.g., NOD-like receptors (NLR)] (9), thereby leading to the activation of various transcription factors, including NF-kB signaling (10) (Figure 1).

Increased physical activity in childhood obesity is an effective intervention to establish a healthy adult lifestyle in reducing the incidence of chronic diseases, such as diabetes (11, 12). In fact, the decline or lack of physical activity in different environments, especially in schools, could contribute to the development of overweight and obesity and their related health problems (13, 14), along with the associated proinflammatory state (15). In this regard, physical activity has been found to be the main intervention to lose or at least maintain body weight in overweight, obese, or severely obese adults, with the corresponding improvement in the cardiorespiratory fitness level (16). The maintenance of a physically active lifestyle in childhood is the basic to prevent or reduce the risk for cardiometabolic diseases and related complications in adulthood (17). Research in high-fat diet (HFD)-induced obese mice age ranged from 4 to 20 weeks has proven that exercise could prevent the obesity-entailed infiltration of inflammatory macrophages into adipose tissue, in support of a reduction in the pro-inflammatory cytokine production (18, 19). Furthermore, the suppression of neutrophil infiltration into fat tissue, along with the inhibition of the expression of elastase or monocyte chemoattractant protein-1 (MCP-1), was also observed with exercise training in this mouse model (20). Of important note, downregulation of inflammatory mediators/cytokines in an *in vivo* rat model of early obesity in response to exercise training has been found (21). In obese children, a meta-analysis has also revealed the beneficial effect of regular physical activity in the modulation of leptin, adiponectin, and interleukin (IL)-6 inflammatory markers (22). Furthermore, regular exercise contributes to the treatment of non-alcoholic fatty liver disease (NAFLD), with a reduction on intrahepatic fat, along with an increase in β-oxidation of fatty acids and a reduction in ROS *via* increased antioxidant enzyme activity (23). Although elevated oxidative stress has been shown to correlate with a deficiency of vitamin D (24) and index of insulin resistance in obese children (25), it is likely that lifestyle interventions including physical activity and dietary modification would improve oxidative status in this population (26) (Figure 1). Thus, this mini-review aims to evaluate the impact of childhood obesity on inflammation and oxidative stress status and the beneficial effects of exercise on these modifications.

## Exercise-Induced Inflammatory Modification in Childhood Obesity

A recent review has reported significant alterations in the quantity and activation of both peripheral and tissue immune cells in obese children, although the obesity-related immune function in childhood still remains to be further investigated due to the limited data from the literature (27). As presented in Table 1.1A, increased levels of C-reactive protein (CRP) and neutrophil count were found to positivity correlate with body fat mass in children and adolescents aged 7–18 years (28, 29). Similarly, Vehapoglu et al. (30) reported increased levels of CRP, white blood cell, and neutrophil count, as well as the neutrophil/lymphocyte ratio, which were positively correlated with BMI and index of insulin resistance, demonstrating the obesity-induced chronic low-grade inflammation in childhood (2–11 years). In agreement with these findings, an increase in the circulating levels of proinflammatory cytokines such as leptin, tumor necrosis factor (TNF)-α, IL-6, and CRP was also observed in obese compared to normal-weight children and adolescents aged 2–18 years (30–39), with a concomitant reduction of adiponectin levels in a similar age group (5–18 years) (34, 37, 40). Additionally, higher levels of plasma hs-CRP, TFN-α, and leptin were reported in both overweight and obese children, while MCP-1 was only found to be higher in the obese than overweight and control subjects with no differences in IL-6 and adiponectin among groups (41). In this regard, obese children (4–14 years) with carbohydrate metabolic impairment have been shown to exhibit an elevation in the concentrations of pro-inflammatory cytokines (e.g., CRP, IL6, TNF-α) compared to controls (36). Importantly, Carolan et al. (34) further examined the phenotype and activation of immune cells and found a shift of pro-inflammatory M1 macrophages as evidenced by elevated soluble CD163 level, along with a greater capacity of peripheral blood mononuclear cells (PBMCs) to produce IL-1β *ex vivo* following lipopolysaccharide (LPS) stimulation in obese *vs.* non-obese children and adolescents aged 6–18 years. A similar observation was also reported by Schipper et al. (42), showing that obese children (6–16 years) elicited an increase in the numbers of classical (CD14^++^CD16^–^) monocytes, as well as the levels of CD11b and IL-6 after LPS treatment compared to non-obese children. Taken together, these findings might partially support the macrophages infiltration and activation associated with obesity-induced inflammation as previously discussed. Additionally, in an interesting study evaluating the levels of several inflammatory cytokines in the saliva of children with dental caries, results showed increased levels of IL-6 and IL-15 in overweight/obese compared to normal-weight children aged 3–8 years (43). In an attempt to evaluate the gender effect of childhood obesity in the circulating levels of inflammatory cytokines, Murdolo et al. (38) showed that obese girls presented elevated IL-8, IL-18, MCP-1, and soluble intercellular adhesion molecule-1 levels (sICAM-1), whereas only obese boys exhibited increased macrophage migration inhibitory factor (MIF) levels. However, although the concentration of appetite-stimulating hormone, adiponectin, was higher in normal-weight girls compared to boys age ranged from 5 to 13 years, no sex by BMI group interaction was found in this study (38). Overall, these outcomes confirm the obesity-induced chronic low-grade inflammation during childhood and propose the elevation of immune cell activation, such as macrophage infiltration, with limited gender influence during puberty.

**TABLE 1:** Effects of childhood obesity (1) and exercise (2) in obese children on inflammation (A) and oxidative stress (B) markers.

1A. Inflammation				

Research article	Age (years)	Subjects	Tissue	Results
Araki et al. (40)	9.9	Obese (*n* = 44; F/M: 16/28) and non-obese (*n* = 28; F/M: 13/15)	Plasma	(↓) Adiponectin
Carolan et al. (34)	12.8 ± 3.2 13.0 ± 3.0	Obese (*n* = 29; F/M: 16/13) Non-obese (*n* = 20; F/M: 7/13)	LPS-stimulated PBMCs supernatant	(↓) CD3^+^ T lymphocytes (↑) sCD163, IL-1β, TNF-α
			Serum	(↑) Leptin (↓) Adiponectin
Chang et al. (41)	9.32 ± 0.45 10.70 ± 0.37 10.06 ± 0.60	Obese (*n* = 19; M) Overweight (*n* = 10; M) Lean (*n* = 16; M)	Whole blood	(↑, ob) hs-CRP, IL-6, MCP-1, Leptin, TNF-α (↑, ow) hs-CRP, IL-6, Leptin, TNF-α (↓, ow) MCP-1
Codoñer-Franch et al. (31)	7–14	Obese (*n* = 60; F/M: 23/37) and non-obese (*n* = 50; FM: 27/23)	Serum	(↑) CRP, IL-6, Leptin, TNF-α
Faienza et al. (35)	10.98 ± 3.2 11.98 ± 3.2 10.48 ± 3.9	Simple obese (*n* = 30; F/M: 16/14) MetS obese (*n* = 25; F/M: 13/12) Normal weight (*n* = 30; F/M: 15/15)	Serum	(↑) CRP
Jaksic et al. (29)	10.83 ± 1.67 11.05 ± 1.45 10.82 ± 1.62	Obese (*n* = 35) Pre-obese (*n* = 82) Normal weight (*n* = 85)	Serum	(↑) CRP (NS) RBP
Lechuga-Sancho et al. (36)	4–14	Obese (*n* = 18; F/M: 10/8) and normal weight (*n* = 6; F/M: 2/4)	Serum	(↑) CRP, IL-6, Leptin (NS) HGF, TNF-α
Mãrginean et al. (32)	10.86 ± 3.5 12.46 ± 3.4	Obese (*n* = 91) Normal weight (*n* = 102)	Serum	(↑) IL-6, Leptin, TNF-α (↓) IL-1β
Murdolo et al. (38)	5–13	Obese (*n* = 140; F/M: 70/70), overweight (*n* = 60; F/M: 28/32) and normal weight (*n* = 105, F/M: 46/59)	Serum	(↑, ob) (M) MIF, (F) IL-8, IL-18, MCP-1, sICAM-1 (↑, ow) (M) Adiponectin (NS, ob, ow) (F) Adiponectin, (M) HMW Adiponectin, (F/M) IP-10, RANTES, Resistin (↑, ob, ow, F, M) Leptin, Leptin/HMW ratio (NS, ob, F) IL-8, IL-18, MCP-1, sICAM-1
Oliver et al. (33)	12.9 ± 0.3	Obese (*n* = 55, F/M: 25/30) and normal weight (*n* = 43, F/M: 25/18)	Plasma	(↑) CRP, IL-6
Ramírez-De Los Santos et al. (43)	5.8	Overweight/obese (*n* = 37) and normal weight (*n* = 43)	Saliva	(↑) IL-6, IL-15 (↓) IL-8, IL-18
Rowicka et al. (39)	7.5	Obese children (*n* = 62)	Serum	(↑) CRP
	6.4	Non-obese children (*n* = 21)		
Schipper et al. (42)	6–16	Obese (*n* = 60; F/M: 33/27) and lean (*n* = 30; F/M: 16/14)	Plasma	(↑) Chemerin, EGF, HGF, IL-8, IL-18, IP-10, Leptin, TIMP-1, TNF-R2
			Monocytes	(↑) CD14^++^CD16^–^ monocytes (NS) CD14^+^CD16^++^monocytes
			LPS-stimulated whole blood	(↑) IL-6 (NS) TNF-α
Singer et al. (28)	13.1	Obese (*n* = 1,207), overweight (*n* = 1,008), underweight (*n* = 130) and normal weight (*n* = 3260)	Serum	(↑) CRP
Vehapoglu (30)	7.4 ± 2.7	Obese (*n* = 90; F/M: 49/41)	Blood	(↑) WBC, NC, NLR (NS) LC
	7.0 ± 2.6	Underweight (*n* = 80; F/M: 39/41)		
	7.2 ± 2.7	Normal weight (*n* = 80; F/M: 41/39)	Serum	(↑) CRP
Woo et al. (37)	11.30 ± 1.17 11.32 ± 1.06	Obese (*n* = 20) Normal weight (*n* = 19, M)	Serum	(↑) Leptin (↓) Adiponectin

**2A. Inflammation and exercise**					

**Research article**	**Age (years)**	**Subjects**	**Training protocol**	**Tissue**	**Results**

Blüher et al. (79)	12.5 ± 0.2	Overweight/obese (*n* = 65)	13-month (150 min/w) endurance and resistance exercise	Serum	(↑) Resistin (↓) Leptin (NS) Adiponectin, CRP, sTNFR-II
Kelly et al. (49)	10.9 ± 0.4	Overweight training (*n* = 10; F/M: 5/5) and control (*n* = 10; FM: 6/4)	8-week (4 d/w) 30–50 min stationary cycling up to 80% VO_2max_	Serum	(NS) CRP
Kelly et al. (50)	10.8 ± 0.67 11.0 ± 0.71	Overweight training (*n* = 9; F/M: 5/4) Overweight control (*n* = 10; FM: 6/4)	8-week (4 d/w) 30 to 50 min stationary cycling up to 80% VO_2max_	Serum	(NS) Adiponectin, CRP, IL-6, Leptin, Resistin, TNF-α
Liu and Timmons (81)	9.5 ± 1.2	Obese training (*n* = 10)	2 × 30 min acute bouts of continuous cycling at 60% VO_2max_	PBMCs	(↑) IL-6, TNF-α
Merlin et al. (15)	9.00 ± 1.96 7.54 ± 1.70	Obese active (*n* = 33) Obese sedentary (*n* = 17)	1-week (7 d/w) daily step count: sedentary (9338 ± 902 steps) *vs.* active (13,614 ± 1,003 steps)	ConA-stimulated lymphocyte supernatant	(↑) IL-2, IL-17, IFN-γ, TNF-α (↓) IL-10 (NS) IL-4, IL-6
Nascimento et al. (47)	10.41 ± 1.96 10.49 ± 2.67	Obese training (*n* = 80; F) Obese control (*n* = 37; F)	8-month (5 d/w) 1 h moderate-to-vigorous aerobic and strength endurance training, flexibility, coordination and balance	Plasma	(↑) Adiponectin (↓) CRP, TNF-α (NS) IL-1β, IL-6, Leptin, Resistin
Nassis et al. (51)	13.05 ± 1.75	Obese (*n* = 19; F)	12-week (3 d/w) 40 min physical training games	Serum	(NS) Adiponectin, CRP, IL-6
Nemet et al. (48)	10.41 ± 1.96 10.49 ± 2.67	Obese training (*n* = 21) Obese control (*n* = 21)	13-week (2 d/w) 1 h team sports and running games	Serum	(↑) Adiponectin (↓) CRP (NS) IL-6, Leptin
Quiroga et al. (52)	10.8 ± 0.3	Obese training (*n* = 25; F/M: 12/13) and control (*n* = 14; F/M: 7/7)	12-week (2 d/w) 1 h combined strength and endurance training	PBMCs	(↓) CASP-1, NLRP3, OPN (NS) TLR4, LPS
Woo et al. (37)	11.32 ± 1.06	Normal weight (*n* = 19)	12-week aerobic training at HRR 45–65%	Serum	(↑, 12-w, 24-w *vs.* basal, ObT, ObD) Adiponectin (↑, 24-w *vs.* 12-w, ObT) Adiponectin (NS, 24-w *vs.* 12-w, ObD) Adiponectin (↓, 12-w, 24-w *vs.* basal, ObT) Leptin (↓, 24-w *vs.* 12-w, ObT) Leptin (NS, 24-w *vs.* 12-w, ObD) Leptin
	11.30 ± 1.17	Obese/overweight training (*n* = 10)	24-week aerobic training at HRR 45–65%		
		Obese/overweight detraining (*n* = 10)	12-week aerobic training at HRR 45–65% + 12-week detraining		

**1B. Oxidative stress**				

**Research article**	**Age (years)**	**Subjects**	**Tissue**	**Results**

Albuali (59)	9.5 ± 1.5	Obese (*n* = 64; F/M: 23/41), overweight (*n* = 83; F/M: 23/60), normal weight (*n* = 66; F/M: 24/42)	Erythrocytes	(↑, ob): AOPPs, MDA, Ox-LDL (↓, ob): Catalase, GSH, GSH-Px, SOD, GSSG (↑, ow): Catalase, GSH, GSH-Px, SOD, GSSG (NS, ow): AOPPs, MDA, Ox-LDL
Araki et al. (40)	9.9	Obese (*n* = 44; F/M: 16/28) and non-obese (*n* = 28; F/M: 13/15)	Plasma	(↑) 8-epi-PGF2α (↓) TAC
Calcaterra et al. (62)	11.8 ± 2.6	Obese (*n* = 53; F/M: 25/28), overweight (*n* = 76; F/M: 41/35) and normal weight (*n* = 49; F/M: 22/27)	Serum	(↑) Ox-LDL
Carmona-Montesinos et al. (65)	4.4 ± 0.12 4.1 ± 0.109	Obese (*n* = 50) Normal weight (*n* = 50)	Plasma	(↑) GSSG, Ox-LDL (↓) GSH
			Serum	(↑) MDA, TBARS
Codoñer-Franch, Boix-García et al. (63)	10.9 ± 2.5	Obese (*n* = 48; F/M: 22/26) and normal weight (*n* = 20; F/M: 8/12)	Plasma	(↑) CG, LPO, MDA (NS) α-tocopherol, β-carotene
			Erythrocytes	(↑) GPX (↓) GSH
Codoñer-Franch, Pons-Morales et al. (64)	10.9 ± 2.5	Obese (*n* = 22; F/M: 12/10) and normal weight (*n* = 16; F/M: 8/8)	Plasma	(↑) CG, LPO, α-tocopherol, β-carotene (NS) MDA
			Erythrocytes	(↑) GPX (NS) GSH
Codoñer-Franch et al. (31)	7–14	Obese (*n* = 60; F/M: 23/37) and non-obese (*n* = 50; FM: 27/23)	Plasma	(↑) 8-isoprostane, AOPPs, MDA
Faienza et al. (35)	10.98 ± 3.2 11.98 ± 3.2 10.48 ± 3.9	Simple obese (*n* = 30; F/M: 16/14) MetS obese (*n* = 25; F/M: 13/12) Normal-weight (*n* = 30; F/M: 15/15)	Plasma	(↑) d-ROMs (↓) BAP, BAP/d-ROMs ratio
Jaksic et al. (29)	10.83 ± 1.67 11.05 ± 1.45 10.82 ± 1.62	Obese (*n* = 35) Pre-obese (*n* = 82) Normal weight (*n* = 85)	Serum	(↑) TAS
Kelly et al. (61)	12.4 ± 3.3 11.7 ± 3.5	Overweight/obese (*n* = 38; F/M: 12/26) Normal weight (*n* = 40; F/M: 15/25)	Serum	(↑) Ox-LDL
Lechuga-Sancho et al. (36)	4–14	Obese (*n* = 18; F/M: 10/8) and normal weight (*n* = 6; F/M: 2/4)	Erythrocytes	(↓) Catalase, GSH, GSSG, Ox-LDL, TAC tGSH
			Serum	(NS) TBARS
			Urine	(↑) 8-isoprostane
Lentferink et al. (67)	11.8 12.2	Obese (*n* = 143; F/M: 70/73) Normal weight (*n* = 428; F/M: 210/218)	Serum	(↑) AGEs
Oliver et al. (33)	12.9 ± 0.3	Obese (n = 55, F/M: 25/30) and normal weight (*n* = 43, F/M: 25/18)	Plasma	(↑) F2-IsoP (NS) SOD, GSH-420
Rowicka et al. (39)	7.5	Obese children (*n* = 62)	Serum	(↑) TOC (↓) TAC (NS) Ox-LDL
	6.4	Non-obese children (*n* = 21)		
Rupérez et al. (66)	3–17	Obese (*n* = 680), overweight (*n* = 358) and normal weight (*n* = 406)	Plasma	(NS, ppb) Ox-LDL (↑, ppb) Retinol (↓, ppb) Carotenes/TAG, Tocopherols/TAG (↓, pb, ppb) TAC
Sfar et al. (60)	6–12	Obese (*n* = 54; F/M: 31/23) and normal weight (*n* = 52; F/M: 25/27)	Erythrocytes	(↑) SOD (NS) Catalase, GPX
Vehapoglu (30)	7.4 ± 2.7	Obese (*n* = 90; F/M: 49/41)	Serum	(↓) TAS, Total thiol (NS) TOS, OSI
	7.0 ± 2.6	Underweight (*n* = 80; F/M: 39/41)		
	7.2 ± 2.7	Normal weight (*n* = 80; F/M: 41/39)		
Woo et al. (37)	11.30 ± 1.17 11.32 ± 1.06	Obese (*n* = 20) Normal weight (*n* = 19, M)	PBMCs	(↑) GPX (NS) SOD

**2B. Oxidative stress and exercise**					

**Research article**	**Age (years)**	**Subjects**	**Training protocol**	**Tissue**	**Results**

Ahmadian et al. (70)	11.4 ± 0.7 11.2 ± 0.7 12.8 ± 0.8 12.5 ± 1.2	Obese asthmatic (*n* = 10; M) Obese non-asthmatic (*n* = 15; M) Lightweight asthmatic (*n* = 10; M) Lightweight non-asthmatic (*n* = 7; M)	Acute progressive aerobic cycle test until volitional exhaustion	Saliva	(↓, all groups) MDA
Dennis et al. (72)	9.3 ± 1.1	Obese high-dose training (*n* = 36), low-dose training (*n* = 34) and control (*n* = 42)	10–15-week (every school day) high-dose (40 min) vs low-dose (20 min) aerobic exercises	Plasma	(NS) F2 isoprostate
Kelly et al. (50)	10.8 ± 0.67 11.0 ± 0.71	Overweight training (*n* = 9; F/M: 5/4) Overweight control (*n* = 10; F/M; 6/4)	8-week (4 d/w) stationary cycling up to 80% of VO_2max_	Serum	(=) 8-isoprostane
Paltoglou et al. (71)	10.95 ± 0.99	Male obese training (*n* = 23) and normal weight training (*n* = 53)	Acute bout of aerobic exercise until exhaustion at 70% of VO_2max_	Erytrocytes	(↑) Catalase (↓) GSH, GSH/GSSG
				Serum	(↑) GPX, PCs, TBARS, TAC
Woo et al. (37)	11.32 ± 1.06	Normal weight (*n* = 19)	12-week aerobic training at HRR 45–65%	Plasma	(↑, 12-w, 24-w *vs.* basal, obT, obD) GPX (NS, 24-w *vs.* 12-w, obT) GPX (↓, 24-w *vs.* 12-w, obD) GPX (NS, 12-w *vs.* basal, obT, obD) SOD (↑, 24-w *vs.* basal, obT, obD) SOD (↑, 24-w *vs.* 12-w, obT) SOD (NS, 24-w *vs.* 12-w, obD) SOD
	11.30 ± 1.17	Obese/overweight training (*n* = 10)	24-week aerobic training at HRR 45–65%		
		Obese/overweight detraining (*n* = 10)	12-week aerobic training at HRR 45–65% + 12-week detraining		
				PBMCs	(↑, 12-w *vs.* basal, Nw, ob) GPX, SOD (↑, 12-w *vs.* basal, ObT, obD) SOD (NS, 12-w *vs.* basal, ObT, obD) GPX (↑, 24-w *vs.* basal, obT) GPX, SOD (NS, 24-w *vs.* basal, obD) GPX, SOD (↑, 24-w *vs.* 12-w, obT) SOD (NS, 24-w *vs.* 12-w, obD) SOD (NS, 24-w *vs.* 12-w, obT, obD) GPX

*(↓), decreased; (↑), increased; (NS), no significant; 8-epi-PGF2α or 8-isoprostane or F2 isoprostate or F2-IsoP, 8-epi-prostaglandin F2 alpha; AGEs, advanced glycation end products; AOPPs, advanced oxidation protein products; BAP, biological antioxidant potential; CASP-1, caspase 1; CD, cluster of differentiation; CD14^++^CD16^–^, classical monocytes; CD14^+^CD16^++^, non-classical monocytes; CG, carbonyl groups; ConA, concanavalin A; CRP, C-reactive protein; d, days; d-ROMs, diacron reactive oxygen metabolites; EGF, epidermal growth factor; F, female; GPX or GSH-Px, glutathione peroxidase; GSH, reduced glutathione; GSSG, oxidized glutathione; h, hour; HGF, Hepatocyte growth factor; HRR, heart rate recovery; hs-CRP, high sensitivity CRP; IFN-γ, interferon-gamma; IL, interleukin; IP-10, IFN-gamma-induced protein 10; LC, lymphocyte count; LPO, lipoperoxides; LPS, lipopolysaccharide; M, male; MCP-1, monocyte chemoattractant protein 1; MDA, malonaldehyde; MIF, macrophage migration inhibitory factor; min, minutes; NC, neutrophil count; NLR, neutrophil/lymphocyte ratio; NLRP3, nod like receptor family pyrin domain containing 3; ob, obese; obD, obese detraining; OPN, osteopontin; obT, obese training; ow, overweight; OSI, oxidative stress index; ox-LDL, oxidized low-density lipoproteins; PBMCs, peripheral blood mononuclear cells; PCs, protein carbonyls; ppb, prepubertal; RANTES, regulated on activation, normal T-cell expressed and secreted; RBP, retinol-binding protein; sCD163, soluble hemoglobin scavenger receptor CD163; sICAM, soluble intercellular adhesion molecule; SOD, superoxide dismutase; sTNFR, soluble tumor necrosis factor receptor; TAC, total antioxidant capacity; TAG, triacylglycerols; TAS, total antioxidant status; TBARS, thiobarbituric acid reactive substances; tGSH, total glutathione equivalents; TIMP-1, TIMP (tissue inhibitor of metalloproteinase) metallopeptidase inhibitor 1; TLR4, toll like receptor 4; TNF-α, tumor necrosis factor-alpha; TOC, total oxidant capacity; TOS, total oxidant status; VO_2max_, maximal oxygen uptake; vs., versus; w, week; WBC, White blood cell.*

Both epidemiologic and longitudinal research have supported that regular exercise or increased physical activity is an effective strategy in reducing systemic low-level inflammation in individuals with obesity and related conditions (44–46). As summarized in Table 1.2A, Nascimento et al. (47) conducted an 8-month longitudinal study in investigating the effects of aerobic exercise training on the inflammatory status in childhood (5–17 years) obesity and observed a reduction in the levels of TNF-α and CRP, along with an improvement in lipid profile and insulin resistance. These findings are partially supported by Nemet et al. (48), showing an increase in adiponectin and a decrease in CRP, along with no changes in leptin and IL-6 levels in obese self-referred children aged 6–13 years following a 3-month combined nutritional-behavioral-physical activity intervention. Interestingly, Woo et al. (37) found a significant increase but a reduction in the serum levels of adiponectin and leptin, respectively, following a minimum of 12 weeks of aerobic training in obese children (the average age of 11 years), despite no change in the percentage of body fat. In contrast, Kelly et al. (49, 50) did not observe any effect of 8 weeks of stationary cycling intervention in plasma CRP, IL-6, TNF-α, adiponectin, and leptin levels, as well as change in weight or body composition in overweight children in the same range of age. Similarly, no significant difference was found in serum adiponectin, IL-6, and CRP levels in overweight and obese girls aged 9–15 years in response to 12-week aerobic training (51). Additionally, although Blüher et al. (51) reported a decrease in serum leptin following the obesity therapy program, including 150 min/week of physical activity for 39 weeks, no change was observed in adiponectin and CRP levels in these overweight/obese children, independently of gender, age, or pubertal stage. Finally, in a recent study examining the effect of a 12-week combined strength and endurance training program in PBMCs of obese pediatric patients aged 7–12 years, a lower NLRP3 inflammasome (a regulator of innate immune activity/pro-inflammatory response) activation, but not TLR4 was reported (52). Similar contradictions have been described in obese adolescents conducting long-term resistance and aerobic exercise programs, showing either decreases or no changes in pro-inflammatory markers such as CRP, IL-6, leptin, or TNF-α (53–58). Additionally, while a reduction in the number of neutrophils and the expression of TNF-α was observed after a 6-month high-intensity exercise, an opposite response was found with the low-intensity protocol (53). Thus, these contradictory findings in the improvement of exercise-mediated inflammation in childhood obesity may be the result of various intensities and/or duration of the intervention, and other factors including dietary changes could also alter these inflammatory profiles.

## Childhood Obesity: Oxidative Stress Status and Benefits of Exercise

Oxidative stress is another underlying mechanism involved in the pathogenesis of obesity-related diseases. Regarding the oxidative status (Table 1.1B), research has demonstrated lower antioxidant activities and higher oxidation products in obese *vs.* non-obese children aged 6–12 years (59). Thus, an increase in total oxidant capacity (TOC) (39) and a decrease in total antioxidant capacity (TAC) (39, 40), as well as a depletion in total antioxidant status (TAS) and total thiols, and a rise in total oxidation status (TOS) (30) were reported in obese children aged 2–13 years compared to their normal-weight counterparts. Moreover, the erythrocyte activities of superoxide dismutase (SOD), catalase, glutathione peroxidase (GPX), glutathione reductase (GR), and reduced glutathione concentration (GSH) were lower in obese children aged 6–12 years than their counterparts, whereas these antioxidant activities in erythrocyte (59) and serum TAS (29) were increased in overweight compared to normal-weight children aged 7–15 years, suggesting the inability to counterbalance the detrimental effects or the highly production of ROS in obesity. The results of this oxidative stress status from these obese children are in agreement with the study by Lechuga–Sancho et al. (36), demonstrating lower erythrocyte levels of catalase, GSH, and TAC in children aged 4–14 years. However, the contradictory findings have been reported including an increase in SOD and no differences in catalase and GPX activity in erythrocytes (60), elevated GPX and no changes in SOD in PBMCs (37), and no differences in plasma SOD and GSH levels (33) between obese and normal-weight children aged 6–12 years. On the other hand, obese children aged 6–12 years exhibited elevated levels of erythrocyte malondialdehyde (MDA) as well as both plasma oxidized low-density lipoproteins (ox-LDL) and advanced oxidation protein products (AOPPs), while no difference was reported in these markers between overweight and normal-weight groups (59). In support of these results, elevated levels of ox-LDL (61, 62) and 8-epi-prostaglandin F2 alpha (31, 33, 36, 40) have been shown to be higher in obese than normal-weight children aged 4–18 years, along with an elevation in circulating levels of MDA, and AOPPs (31). Importantly, Codoñer–Franch et al. (63) have previously demonstrated an increase in plasma MDA and carbonyl groups (CG), as well as erythrocyte GPX activity levels, along with decreased levels in erythrocyte GSH, in severely obese children only, whereas the levels of α-tocopherol and β-carotene, lipophilic antioxidants that act in biomembranes as scavengers of free radicals, remained similar as obese and normal-weight children aged 6–14 years. Although another investigation by Codoñer–Franch et al. (64) showed that the levels of plasma lipoperoxides (LPO), CG, and the antioxidants α-tocopherol and β-carotene, as well as the GPX erythrocyte activity were increased in obese children in the same range of age, plasma MDA levels and erythrocyte GSH concentration did not differ between groups. Similar results have been presented in obese infants aged 3–5 years, showing higher serum peroxidized lipids, specifically thiobarbituric acid-reacting substances (TBARS), plasmatic ox-LDLs and oxidized glutathione (GSSG) levels, as well as lower plasmatic GSH values, compared with their matched controls (65). Thus, the inconsistence of the above findings could potentially be due to the wide range of age groups utilized in these studies. To further clarify this potential effect of age, Rupérez et al. (66) differentiated children aged 3–17 years during pre/pubertal period and found that TAC levels were lower in prepubertal but higher in pubertal overweight/obese children than normal-weight children. Furthermore, the advanced glycation end products (AGEs) formation (a facilitator of ROS), measured as skin autofluorescence, was increased in obese children and adolescents aged 4–18 years, although no differences were shown in the group of children under 10 years old (67). These observations might be partially supported by the transient decline in insulin sensitivity during puberty attributable to lack of glycemic control (68, 69). In contrast, a high level of serum ox-LDL observed in obese children (the mean age = 11 years) was not correlated with sex, age, and pubertal status (62). It is reasonably speculated that the metabolic status and the presence of the metabolic syndrome could have influenced these reported results, as evidenced by Rupérez et al. (66) with lower carotenes and tocopherols levels in metabolically unhealthy *vs.* healthy children aged 3–17 years. In this regard, Faienza et al. (35) presented similar results, demonstrating a higher level of diacron reactive oxygen metabolites (d-ROMs) in obese children and prepubertal subjects (the average age of 11 years) with metabolic syndrome, with a negative relationship between total antioxidant capacity and standardized BMI. Thus, these findings suggest that an early onset in the pathogenesis of obesity-mediated diseases by elevated oxidative stress may occur during pediatric age.

Although there is limited research investigating the impact of physical activity interventions on childhood obesity-associated oxidative stress (Table 1.2B), acute aerobic cycling exercise seems to exert contrary effects on oxidative markers in obese children. Specifically, Ahmadian et al. (70) reported a decrease in MDA levels after an acute progressive test until volitional exhaustion in both obese and normal-weight children under the age of 13 years, as well as in their asthmatic counterparts (70), Paltoglou et al. (71) reported an increase in the levels of TBARS and protein carbonyls (PCs), as well as in TAC, and catalase, along with a decrease in the production of GSH and GSH/GSSG in both prepubertal and pubertal obese children following an acute bout of maximal aerobic exercise. These responses to acute aerobic exercise were also observed in their prepubertal and pubertal normal-weight counterparts. On the other hand, neither an 8-week nor 13-week aerobic training program improved the levels of plasma 8-isoprostane (50) or serum F2-isoprostane (72) in overweight/obese children aged 7–11 years. Contrary to these results, a 12-week training exercise was effective to increase both SOD and GPX mRNA expression in PBMCs from both obese and normal-weight children (the mean age = 11 years) (37). Interestingly, this study reported that the expression of SOD and GPX mRNA remained elevated until completion of a 24-week training intervention in the obese training group, but not the obese detraining group who participated in the 12-week training plus 12-week detraining program. Similarly, an elevation in plasma levels of SOD and GPX following 24 weeks of exercise program in both obese training and detraining groups was observed, whereas only obese detraining group showed a decrease in GPX and no changes in SOD level at the mid-training (12 weeks) (37). The phenomenon of this observation might present a different behavior or time course of these antioxidant enzyme responses, depending on the oxidation-reduction transcription factors involved in the regulatory processes. Overall, these results indicate that chronic exercise could possibly elicit protective adaptations against oxidative damage in childhood obesity as the effective training interventions have been administered in adult obesity (73, 74).

Finally, oxidative stress is frequently associated with obesity-induced inflammation. Although the mechanisms for childhood obesity-induced oxidative stress remain to be investigated, leptin has been proposed as an important contributor, due to its role in mediating pro-inflammatory state in obese individuals (75, 76). Particularly, increased leptin has been shown to modulate the production of oxidative stress biomarkers, such as reduced nitric oxide (NO), increased superoxide (O2^–^) and peroxynitrite (ONOO-) in both human endothelial cells and the endothelium of obese mice (77, 78). With a reduction in the circulating level of leptin following training in children and adolescences aged 7–18 years (79) and its potential relationship with oxidative stress (80), this could partially support the role of exercise interventions for oxidative adaptions in alleviating susceptibility to obesity-associated oxidative stress.

## Conclusion

Results from this mini-review evidence that localized inflammation in adipose tissue triggers low-grade systemic inflammation in childhood obesity. Similarly, an increase in oxidative stress markers, as well as a decrease in antioxidant markers in obese children present an imbalance in oxidative stress status. However, the effects of physical activity on the inflammatory and oxidative stress responses in growing obese kids still remain inconclusive. Regarding oxidative status, although the studies available to date are not sufficient to conclude the beneficial effect of exercise in obese children, the influence of puberty in childhood obesity-mediated oxidative stress is warranted for further investigation. Moreover, the research addressing the role of several key hormones (e.g., testosterone, estrogen, and growth hormone) on the obesity-associated inflammatory and oxidative stress processes in the different growth stages of children is needed to further discover their exercise modulation, including a limited age range with controlling for biological and chronological age. In summary, additional insight into how physical activity interventions influence the cellular adaptations of inflammation and oxidative stress is necessary to better understand the importance of exercise as an antagonist to the current childhood obesity epidemic.

## Author Contributions

BE, MJC, and JG-G conceptualized the manuscript. BE and MR-V performed the literature search. BE, C-JH, and MJC performed manuscript drafting. MJC and JG-G supervised and revised the manuscript. All authors contributed to the article and approved the submitted version.

## Conflict of Interest

The authors declare that the research was conducted in the absence of any commercial or financial relationships that could be construed as a potential conflict of interest.

## Publisher’s Note

All claims expressed in this article are solely those of the authors and do not necessarily represent those of their affiliated organizations, or those of the publisher, the editors and the reviewers. Any product that may be evaluated in this article, or claim that may be made by its manufacturer, is not guaranteed or endorsed by the publisher.

## Abbreviations

8-epi-PGF2 α or 8-isoprostane or F2 isoprostate or F2-IsoP: 8-epi-prostaglandin F2 alpha
AGEs: advanced glycation end products
AOPPs: advanced oxidation protein products
BAP: biological antioxidant potential
BMI: body mass index
CASP-1: caspase 1
CG: carbonyl groups
CD: cluster of differentiation
CD14^++^CD16^–^: classical monocytes
CD14^+^CD16^++^: non-classical monocytes
ConA: concanavalin A
CRP: C-reactive protein
d: days
d-ROMs: diacron reactive oxygen metabolites
EGF: epidermal growth factor
F: female
GPX or GSH-Px: glutathione peroxidase
GSH: reduced glutathione
GSSG: oxidized glutathione
HGF: Hepatocyte growth factor
HRR: heart rate recovery
hs-CRP: high sensitivity CRP
IFN- γ: interferon-gamma
IL: interleukin
IP-10: IFN- γ -induced protein 10
LC: lymphocyte count
LPO: lipoperoxides
LPS: lipopolysaccharide
M: male
MCP-1: monocyte chemoattractant protein 1
MDA: malonaldehyde
MIF: macrophage migration inhibitory factor
NC: neutrophil count
NF-kB: nuclear factor NF-kappa-B
NLR: neutrophil/lymphocyte ratio
NLRP3: nod like receptor family pyrin domain containing 3
ob: obese
OPN: osteopontin
ow: overweight
OSI: Oxidative stress index
ox-LDL: oxidized low-density lipoproteins
pb: pubertal
PBMCs: peripheral blood mononuclear cells
PCs: protein carbonyls
ppb: prepubertal
RANTES: regulated on activation, normal T-cell expressed and secreted
RBP: retinol-binding protein
sCD163: soluble hemoglobin scavenger receptor CD163
sICAM: soluble intercellular adhesion molecule
SOD: superoxide dismutase
sTNFR: soluble tumor necrosis factor receptor
TAC: total antioxidant capacity
TAG: triacylglycerols
TAS: total antioxidant status
TBARS: thiobarbituric acid reactive substances
tGSH: total glutathione equivalents
TIMP-1: metalloproteinase inhibitor 1
TLR4: toll like receptor 4
TNF- α: tumor necrosis factor-alpha
TOC: total oxidant capacity
TOS: Total oxidant status
VO_2max_: maximal oxygen uptake
*vs.*: versus
w: week
WBC: white blood cell.
